# A novel missense *ALMS1* variant causes aberrant splicing identified in a cohort of patients with Alström syndrome

**DOI:** 10.3389/fgene.2022.1104420

**Published:** 2023-01-04

**Authors:** Jie Shi, Ke Xu, Xin Zhang, Yue Xie, Haoyu Chang, Yang Li

**Affiliations:** Beijing Ophthalmology and Visual Sciences Key Lab, Beijing Tongren Eye Center, Beijing Institute of Ophthalmology, Beijing Tongren Hospital, Capital Medical University, Beijing, China

**Keywords:** *ALMS1* gene, Alström syndrome, retinal dystrophy, pre-mRNA splicing, missense variant

## Abstract

**Purpose:** Alström syndrome (AS) is a rare autosomal recessive disorder caused by variants of *ALMS1*. The objectives of this study were to describe the clinical and genetic characteristics of 19 Chinese patients with biallelic variants in *ALMS1*.

**Methods:** We recruited 19 probands with biallelic disease-causing *ALMS1* variants. All patients underwent ophthalmic and systematic evaluations and comprehensive molecular genetic analysis. Reverse transcriptase-polymerase chain reaction (RT-PCR) assays were performed to observe the effect of a novel missense variant on *ALMS1* pre-mRNA splicing.

**Results:** We identified 33 causative variants in *ALMS1*, including 15 frameshift small indels, 14 non-sense variants, two gross deletions, one splicing variant, and one missense variant. RT-PCR showed that the missense variant c.9542G>A (p.R3181Q) altered pre-mRNA splicing to generate a truncated protein p. (Ser3082Asnfs*6). Retinal dystrophy (RD) was noted in all the patients, followed by metabolism disturbance (obesity or acanthosis nigricans) in 66.7% and hearing impairment in 61.1% of the patients. Patient systemic symptom numbers and their age at evaluation showed a significant positive correlation, and BCVA and age at the last examination showed a moderate correlation. All patients exhibited early-onset RD and severe visual impairment. The exception was one patient carrying homozygous p. R3181Q, who showed a mild visual defect and atypical retinal phenotype.

**Conclusion:** Our findings expand the pathogenic variant spectrum of *ALMS1* and provide the first verification of a novel missense variant caused AS by aberrant pre-mRNA splicing. Patients with AS might demonstrate varied clinical spectra; therefore, genetic analysis is vital for the early and accurate diagnosis of patients with atypical AS.

## 1 Introduction

Alström syndrome (AS; MIM# 203800) is a rare multiple-organ disorder with a global prevalence of less than 1 in 1,000,000 (Marshall et al., 2011). AS is inherited in an autosomal recessive pattern and is characterized by retinal dystrophy (RD), sensorineural hearing loss, truncal obesity, insulin resistance with hyperinsulinemia, type 2 diabetes mellitus (T2DM), dilated cardiomyopathy (DCM), and hepatic, renal, and pulmonary dysfunction (Marshall et al., 2005; Marshall et al., 2007a). Many of its cardinal features evolve gradually from childhood; consequently, Marshall et al. (2007a) were able to establish clinical diagnostic criteria based on major and minor criteria stratified by age. The major diagnostic features include vision defects and a monoallelic variant in *ALMS1* or a family history of AS, while the minor diagnostic features comprise disturbances in metabolism, hearing, and heart, liver, and kidney function related to AS. An AS diagnosis can be established by the presence of one major and two minor diagnostic features in infants and toddlers (0–2 years), one major and three minor criteria in children aged 3–14 years, and one major and four minor diagnostic features in patients over 15 years of age (Marshall et al., 2007a). However, a diagnosis of AS can be established at any age when biallelic variants in *ALMS1* are detected in the patient (Marshall et al., 2007a).

The *ALMS1* gene, located on chromosome 2p13.1, is the only disease-causing gene for AS (Collin et al., 2002; Hearn et al., 2002). The gene consists of 23 coding exons and has several transcript isoforms. The longest *ALMS1* transcript encodes ALMS1, a 461 kDa protein composed of 4,169 amino acids; ALMS1 is localized in centrosomes and the base of cilia (Collin et al., 2002; Hearn et al., 2002). ALMS1 is ubiquitously expressed, but its expression levels differ considerably among tissues and organs. It is highly expressed in the testis, moderately expressed in the brain, eye, lung, and olfactory bulb, and minimally expressed in the spleen, liver, and kidney (Collin et al., 2002; Hearn et al., 2002). The biological function mechanism remains unclear; however, ALMS1 is an assumed constituent of actin filaments, with involvement in cilia function, intracellular trafficking, cell differentiation, and maintenance of metabolic homeostasis (Hearn et al., 2005; Knorz et al., 2010; Hearn, 2019). At present, 387 *ALMS1* variants have been recorded according to the Human Gene Variant Database (HGMD) Professional 2021.4. Most variants are located in exons 8, 10, and 16, making these locations variant hotspots (Marshall et al., 2007b; Marshall et al., 2015; Ozantürk et al., 2015; Bea-Mascato et al., 2021). The majority of the *ALMS1* variants are truncating variants, such as non-sense and frameshift indels, and they introduce a premature termination codon (PTC), causing premature protein truncation and triggering a non-sense-mediated decay (NMD) process (Marshall et al., 2005; Marshall et al., 2007a; Marshall et al., 2007b; Marshall et al., 2011; Marshall et al., 2015; Ozantürk et al., 2015; Bea-Mascato et al., 2021). By contrast, only 19 missense variants have been recorded, but their pathogenicity remains unclear (Marshall et al., 2007b; Marshall et al., 2015).

Several recent studies have identified biallelic *ALMS1* variants in patients with Leber congenital amaurosis (LCA) or LCA-like RD, as well as with early-onset severe cone-rod dystrophy (CORD), as RD was usually the first and only clinical symptom of AS appearing in infants or toddlers (Khan et al., 2015; Wang et al., 2015; Xu et al., 2016; Xu et al., 2020). At present, no discernible genotype-phenotype correlation has been established in AS, and one early study even indicated that patients with variants in exon 8 might have delayed and mild renal complications (Marshall et al., 2007b).

The current study examined the genetic and clinical characteristics of 19 probands with biallelic variants in *ALMS1*. Our RNA analysis revealed that one novel missense variant resulted in abnormal *ALMS1* pre-mRNA splicing. We found that the patient with this missense variant exhibited an atypical retinal phenotype with mild visual defects.

## 2 Subjects and methods

### 2.1 Patients

This study was approved by the Beijing Tongren Hospital Joint Committee on Clinical Investigation and was conducted in accordance with the tenets of the Declaration of Helsinki. Informed written consent was acquired from all patients or their guardians before enrollment in the investigation. In total, 19 probands carrying biallelic *ALMS1* variants were recruited from the Genetics Laboratory of the Beijing Institute of Ophthalmology from January 2012 to September 2022. In this cohort, 5 patients had been reported in our previous research (Liu et al., 2009; Xu et al., 2020).

Most of the probands, if they could cooperate, underwent routine ophthalmic assessment, including best-corrected visual acuity (BCVA) using standard Snellen charts, slit-lamp biomicroscopy, fundus photography, and optical coherence tomography (OCT, Spectralis, Heidelberg Engineering, Heidelberg, Germany). Some patients also underwent fundus autofluorescence (FAF, Spectralis, Heidelberg Engineering, and Heidelberg, Germany). Full-field electroretinography (ERG, Roland, Germany) was performed on 13 patients according to the standard protocol of the International Society for Clinical Electrophysiology of Vision (ISCEV). All ophthalmic images and ERG were assessed by one junior and one senior ophthalmology doctor.

All patients underwent general and metabolic examinations, including pure tone audiogram (PTA); height and weight measurements to calculate body mass index (BMI); skin, endocrine, hepatic, and renal function workups; and echocardiography. One exception was patient 067280. The results were evaluated by respective specialists.

### 2.2 PCR-based sequencing of the *ALMS1* gene

We collected peripheral blood samples from all subjects and their available relatives. We then extracted genomic DNA from peripheral blood leukocytes using a genomic DNA extraction kit (Cwbio, Beijing, China), following the manufacturer’s protocol. Exons 8, 10, and 16, as well as their flanking splice sites, of the *ALMS1* gene, were amplified by PCR in 5 patients who were clinically diagnosed with AS. The PCR assays were performed using the 15 pairs of primers listed in Supplementary Table S1 and conventional reaction mixtures. The resulting target fragments were then purified and directly sequenced on an ABI Prism 373A DNA sequencer (Applied Biosystems, Foster City, CA). The results were compared with the known sequence of the *ALMS1* gene (GenBank NM_ 015120.4).

### 2.3 Targeted or whole exome sequencing and bioinformatic analysis

We performed TES in 10 probands using an inherited retinal degeneration (IRD) capture panel and WES in 4 patients. The capture panel design, Illumina library preparation, and capture experiment were performed as previously described (Sun et al., 2018). The raw data were deposited at the Sequence Read Archive (SRA) and could be accessed with NIH BioProject number SUB12390967. We used the HGMD database (http://www.hgmd.cf.ac.uk/ac/index.php, 2020.4) and the LOVD database (https://databases.lovd.nl/shared/genes, 2021.4) to search for reported pathogenic variants. The pathogenicity of the variants was predicted by a panel of *in silico* programs, including Mutation Taster (MT, http://www.mutationtaster.org), PolyPhen2 (PP2, http://genetics.bwh.harvard.edu/pph2/), and SIFT in the public domain, 2021.12. The programs NetGene2 Server (http://www.cbs.dtu.dk/services/NetGene2/), Alternative Splice Site Predictor (ASSP, http://wangcomputing.com/assp/index.html), Berkeley *Drosophila* Genome Project (BDGP, http://www.fruitfly.org/seq_tools/splice.html) were used to analyze variants involving a potential splicing effect. We also performed cosegregation analysis in all families when the DNA of any family members was available.

### 2.4 Reverse transcriptase-polymerase chain reaction analysis

We collected fresh peripheral blood (4 ml) from proband 010737, the proband’s mother, and an unrelated control subject and then separated mononuclear cells (lymphocytes) from peripheral blood samples using a human peripheral lymphocyte separation medium (Suzhou Meilun Biotechnology Co., Ltd.). Total RNA was then extracted from the lymphocytes using an RNAprep Pure Cell/Bacteria Kit (Tiangen, Beijing, China). Reverse transcription was performed with FastKing cDNA Dispelling RT SuperMix (Tiangen, Beijing, China) to synthesize single-stranded cDNA from 1 µg RNA. The reverse-transcribed PCR reaction included an initial step at 42°C for 15 min, followed by 95°C for 3 min. The cDNA was amplified using specific primers for *ALMS1* and *GAPDH* (internal control) (Supplementary Table S2). The PCR fragments were electrophoresed in 2% agarose gels. Each DNA band was confirmed by Sanger sequencing, and the results were assembled with the *ALMS1* transcript NM_015120.4.

### 2.5 Statistical analysis

We converted the decimal visual acuity into the logarithm of the minimum angle of resolution (logMAR) values for statistics. The logMAR values of 0, 1.0, 1.85, 2.3, 2.7, and 3.0 are equal to a Snellen decimal vision of 1.0, 0.1, counting fingers, hand movements, light perception, and no light perception, respectively. The Shapiro-Wilk test was employed to evaluate whether a single group of data conformed to a normal distribution. The Spearman’s correlation coefficient was used to analyze bivariate relationships. We chose BCVA of the right eye for each patient to perform the statistical analysis after inter-eye correlation analysis. All statistical analyses were performed using SPSS Statistics 24.0 (IBM, Armonk, NY, United States of America). A value of *p* < 0.05 was considered statistically significant.

## 3 Results

### 3.1 Genetic findings

**FIGURE 1:**
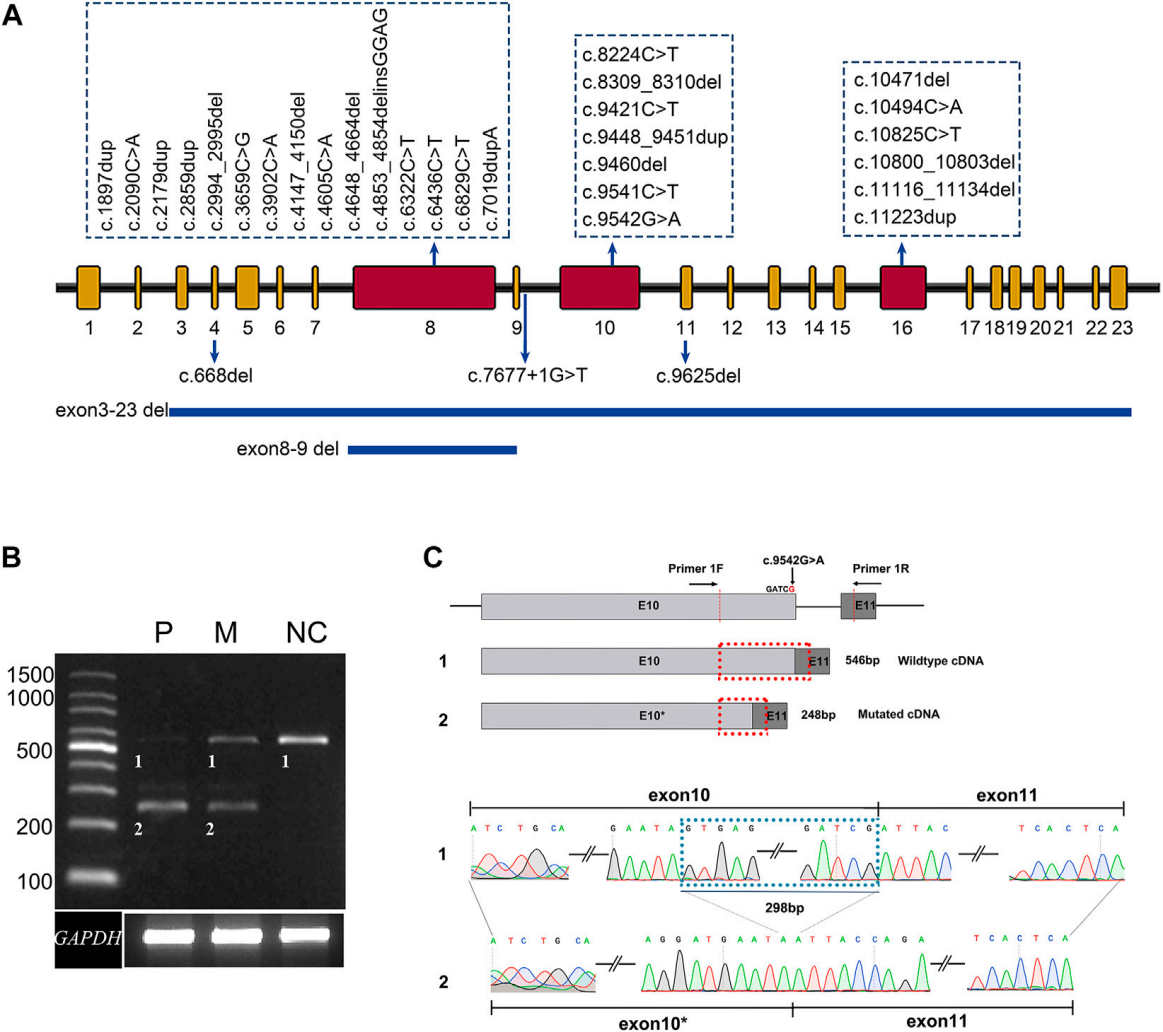
Summary of the variants identified in this study. **(A)** The distribution of 33 distinct variants of *ALMS1* in this study. **(B)** Agarose gel electrophoresis displays the RT-PCR products for ALMS1 mRNA transcripts and GAPDH from the lymphocytes of patient 010737 (P), the patient’s mother (M), and a normal control (NC). **(C)** A schematic diagram shows the position of the novel variant and the cryptic donor site. A sequence chromatogram of the wild-type and aberrant transcripts show a lack of the last 298 bp of exon 10 in the variant.

We identified 33 distinct disease-causing variants of *ALMS1* in the 19 probands. These variants consisted of 15 frameshift indels, 14 non-sense variants, two gross deletions, one splicing effect, and one missense variant. Over 80% of the variants were located in exon 8, exon 10, and exon 16 (Figure 1A), and 13 of the variants identified in the current study were novel (Table 1). Among these 33 variants, variant p. (Arg3609*) was detected three times; the remaining variants were either identified once (29/33) or twice (3/33) (Table 1). None of the 13 novel variants were recorded in any public database, and they were defined as pathogenic based on the American College of Medical Genetics and Genomics (ACMG) guidelines and standards, except for the low-frequency missense variant c.9542G>A, p. (Arg3181 Gln) (Table 1).

**TABLE 1:** Presumed pathogenic *ALMS1* variants identified in this study and analysis of the variants by predictive programs.

Exon	Nucleotide change	Protein effect	Allele#	MT	ASSP	BDGP	Net-Gene2	1000G	ExAC	ACMG	Source
4	c.668del	p. (Ala223Valfs*8)	1	DC	—	—	—	—	—	P	Novel
8	c.1897dup	p. (Tyr633Leufs*9)	1	DC	—	—	—	—	—	P	Marshall et al. (2015)
8	c.2090C>A	p. (Ser697*)	1	DC	—	—	—	—	—	P	Rethanavelu et al. (2020)
8	c.2179dup	p. (Tyr727Leufs*12)	1	DC	—	—	—	—	—	P	Marshall et al. (2007b)
8	c.2859dup	p. (Asn954*)	1	DC	—	—	—	—	—	P	Novel
8	c.2994_2995del	p. (Val999Thrfs*8)	1	DC	—	—	—	—	—	P	Xu et al. (2020)
8	c.3659C>G	p. (Ser1220*)	1	DC	—	—	—	—	-	P	Liu et al. (2009)
8	c.3902C>A	p. (Ser1301*)	1	DC	—	—	—	—	1.16E-04	P	Rethanavelu et al. (2020)
8	c.4147_4150del	p. (Ser1383Asnfs*19)	1	DC	—	—	—	—	—	P	Astuti et al. (2017)
8	c.4605C>A	p. (Tyr1535*)	1	DC	—	—	—	—	—	P	Marshall et al. (2015)
8	c.4648_4664del	p. (Arg1550Trpfs*4)	1	DC	—	—	—	—	—	P	Xu et al. (2020)
8	c.4853_4854delinsGGAG	p. (Ser1618Trpfs*18)	1	DC	-	-	-	-	—	P	Novel
8	c.6322C>T	p. (Gln2108*)	1	DC	—	—	—	—	—	P	Xu et al. (2020)
8	c.6436C>T	p. (Arg2146*)	1	DC	—	—	—	—	8.00E-06	P	Marshall et al. (2015)
8	c.6829C>T	p. (Arg2277*)	2	DC	—	—	—	—	—	P	Marshall et al. (2015)
8	c.7019dupA	p. (Thr2341Aspfs*10)	1	DC	—	—	—	—	—	P	Novel
Intron 9	c.7677 + 1G>T	p. (Gly2558Valfs*2, = )	1	DC	SC	SC	SC	—	—	P	Marshall et al. (2015)
10	c.8224C>T	p. (Gln2742*)	2	DC	—	—	—	—	—	P	Ozantürk et al. (2015)
10	c.8309_8310del	p. (Ser2770Phefs*6)	1	DC	—	—	—	—	—	P	Novel
10	c.9421C>T	p. (Gln3141*)	1	DC	—	—	—	—	—	P	Novel
10	c.9448_9451dup	p. (Ser3151Lysfs*2)	1	DC	—	—	—	—	—	P	Novel
10	c.9460del	p. (Val3154*)	1	DC	—	—	—	—	—	P	Marshall et al. (2015)
10	c.9541C>T	p. (Arg3181*)	1	DC	—	—	—	—	—	P	Rethanavelu et al. (2020)
10	c.9542G>A	p. [Ser3082Asnfs*6, Arg3181 Gln]	2	DC	SC	SC	SC	-	8.40E-06	P	Novel
11	c.9625del	p. (Ser3209Valfs*23)	1	DC	—	—	—	—	—	P	Novel
16	c.10471del	p. (Leu3491Tyrfs*18)	1	DC	—	—	—	—	—	P	Xu et al. (2020)
16	c.10494C>A	p. (Cys3498*)	1	DC	—	—	—	—	—	P	Novel
16	c.10800_10803del	p. (Glu3601Cysfs*60)	1	DC	—	—	—	—	—	P	Rethanavelu et al. (2020)
16	c.10825C>T	p. (Arg3609*)	3	DC	—	—	—	—	—	P	Rethanavelu et al. (2020)
16	c.11116_11134del	p. (Arg3706Leufs*11)	1	DC	—	—	—	—	1.70E-05	P	Rethanavelu et al. (2020)
16	c.11223dup	p. (Glu3742Argfs*4)	1	DC	—	—	—	—	—	P	Novel
—	exon8-9 del	—	1	—	—	—	—	—	—	P	Novel
—	exon3-23 del (208 kb)	—	1	—	—	—	—	—	—	P	Novel

Notes: ACMG, american college of medical genetics; DC, disease causing; ExAC: exome aggregation consortium; P, pathogenic; SC, splice site changed.

### 3.2 Transcript analysis

The pathogenicity of variant c.9536G>A p. (Arg3181 Gln) was predicted as benign, probably damaging, and tolerated by the MT, PP2, and SIFT software, respectively, but the variant was predicted to affect normal splicing of exon 10 by the BDGP, ASSP, and NetGene2 software (Table 1). A further RT-PCR experiment using the lymphocyte cDNA to investigate the variant effect on *ALMS1* pre-mRNA splicing revealed that this variant resulted in a 298 bp truncation of exon 10 due to the introduction of a cryptic donor site in that exon (Figures 1B,C). The 298 bp truncation caused a frameshift coding p. (Ser3082Asnfs*6). In addition, a normal splicing band was weakly detected in patient 010737, who carried a homozygous variant p. (Arg3181Gln) (Figure 1B).

### 3.3 Clinical characteristics

In the current cohort, 19 patients (13 males and 6 females) harbored biallelic disease-causing *ALMS1* variants, as confirmed by cosegregation analyses. All the probands were of Han nationality. Among the patients, seven were clinically diagnosed with CORD, six with AS, five with LCA, and one with Bardet-Biedl syndrome (BBS) (Table 2). The patients’ median age at the last examination was 7.0 years (range: 1.40–30.0). All patients presented with ocular symptoms (100%), followed by endocrine or/and metabolism problems (overweight/obesity, acanthosis nigricans, and T2DM/impaired glucose tolerance) occurred in 12 patients (66.7%), hearing impairment in 11 patients (61.1%), and hepatic dysfunction in 6 patients (33.3%) (Table 3).

**TABLE 2:** The Clinical features and results of the *ALMS1* gene variants screening of the patients.

Patient ID	Gender	Age (years)		BCVA (log MAR) OD/OS	Initial sy-mptom	NY	ERG	Endocrine/Metabolism			Hearing loss	Hepatic dys-function	Renal dys-function	Cardi-ology	Variants	Clinical diag-nosis	Method
		Onset*	Exam					BMI	AN	T2DM/IGT					Allele1/Allele2		
010648	M	0.3	1.4	NA	NY, PP	Y	Cone SD	NA	N	N	N	N	N	N	p.N954*/p.R2146*	CORD	T
P2	M	1.0	2.0	NA	PP	Y	SD	NA	N	N	N	N	N	N	p.R3609*/p.S2770Ffs*6	LCA	T
P1	M	0.3	2.2	NA	NY, PP	Y	NA	21	N	N	N	N	N	N	p.R2277*/p.E3742Rfs*4	CORD	W
010235	M	1.0	3.8	NA	PP, RV	Y	SD	NA	N	N	N	N	N	N	p.Q3141*/p.Y727Lfs*12	CORD	T
067280 †	M	1.0	5.5	0.60/0.60	PP, RV, SQ	Y	EX	NA	NA	NA	NA	NA	NA	NA	p.S1301*/p. (G2558Vfs*2, = )	LCA	T
010621	M	1.0	5.8	NA	PP	NA	SD	22.7	Y	N	N	N	N	N	p.R2277*/p.R3706Lfs*11	CORD	T
010131 †	M	0.8	6.0	1.70/1.40	NY, PP	Y	NA	NA	N	N	Y	N	N	N	p.V999Tfs*8/p.R3181*	LCA	T
010809	F	2.0	6.5	0.92/0.82	RV, PP	N	Cone SD, Rod MoD	NA	N	N	N	N	N	N	p.A223Vfs*8/exon8-9del	CORD	W
0191309	F	0.6	6.8	NA	PP	Y	NA	29.0	Y	N	Y	Y	N	N	p.C3498*/p.S1618Wfs*18	CORD	T
010159 †	F	0.3	7.0	2.70/2.70	NY, PP	Y	EX	NA	Y	N	Y	N	N	N	p.Q2108*/p.R1550Wfs*4	LCA	T
067340 †	M	0.5	7.0	2.70/2.70	PP, RV	Y	SD	NA	Y	N	Y	N	N	N	p.L3491Yfs*18/p.R3609*	LCA	T
010647	F	0.3	7.0	1.30/1.30	PP, RV	Y	SD	21.7	Y	Y	Y	N	N	N	p.R3609*/p.S3209Vfs*23	AS	T
078003 †	M	NA	9.0	1.00/1.00	PP, RV	Y	NA	30.2	Y	Y	Y	Y	Y	N	p.Q2742*, *Homo*	AS	S
0191552	M	0.3	11.0	1.52/1.52	PP	Y	EX	27.9	Y	N	NA	N	N	Y	p.Y633Lfs*9/p.S697*	AS	S
010737	M	6.0	12.3	0.30/0.30	RV	N	Cone SD, Rod MoD	24.2	Y	Y	Y	Y	Y	N	p. [S3082Nfs*6, R3181Q], *Homo*	CORD	W
078021	F	FC	13.0	2.70/2.70	RV, SQ	Y	EX	21.1	Y	Y	Y	Y	N	N	p.S3151Kfs*2/p.V3154*	AS	S
078030	M	1.0	17.0	2.70/2.30	RV	NA	NA	NA	Y	Y	Y	Y	Y	N	p.S1220*/p.Y1535*	AS	S
078015	M	FC	23.3	3.00/3.00	PP, RV	Y	NA	25.6	Y	Y	Y	Y	N	N	p.S1383Nfs*19/exon3-23 del	AS	S
0191834	F	1.0	30.0	3.00/3.00	RV	Y	EX	24.6	N	Y	Y	N	Y	N	p.T2341Dfs*10/p.E3601Cfs*60	BBS	W

Notes: AN, acanthosis nigricans; AS, Alström syndrome; BBS, Bardet-Biedl syndrome; BCVA, best corrected visual acuity; BMI, body mass index; CORD, Cone-rod dystrophy; EX, extinguish; F, female; FC, from childhood; *Homo*, Homozygous; IGT, impaired glucose tolerance; LCA, leber congenital amaurosis; M, male; MoD, moderate damage; N, no; NA, not available; NY, nystagmus; OD, right eye; OS, left eye; Onset*, The onset age of ocular symptom; PP, photophobia; RV, reduced vision; S, sanger sequencing; SD, severe damage; SQ, squint; T, targeted exome sequencing; T2DM, Type 2 diabetes mellitus; W, whole exome sequencing; Y, yes; †, Patients reported in our previous published study.

**TABLE 3:** Presenting symptoms in the patients with AS.

Symptoms	N/T	%	Youngest (years)
Eye	19/19	100	0.3
Reduced vision	19/19	100	0.3
Photophobia	16/19	84.2	0.3
Nystagmus	16/18	88.2	0.3
Squint	2/19	10.5	1.0
Endocrine/Metabolism	12/18	66.7	5.8
Acanthosis nigricans	11/18	61.1	5.8
T2DM/IGT	7/18	38.9	7.0
Hypogonadism	3/18	16.7	7.0
Hyperlipidemia	2/18	11.1	12.3
Overweight/obesity	6/10	60	6.8
Ear	11/18	61.1	6.0
SNHL	11/18	61.1	6.0
Middle ear mastoiditis	3/18	16.7	12.3
Hepatic disfunction	6/18	33.3	6.8
Elevated liver transaminase	6/18	33.3	6.8
Renal disfunction	4/18	22.2	9.0
Proteinuria	1/18	5.6	9.0
Hyperuricemia	3/18	16.7	12.3
Others	3/18	22.2	11.0
Endocardial fibroelastosis	1/18	5.6	11.0
Cognitive impairment	1/18	5.6	13.0
Scoliosis	1/18	5.6	12.3
Brachydactyly	1/18	5.6	0

Notes: IGT, impaired glucose tolerance; N, number of patients with corresponding symptoms; SNHL, sensory neural hearing loss; T, total number of patients with recordings; T2DM, Type 2 diabetes mellitus.

**FIGURE 2:**
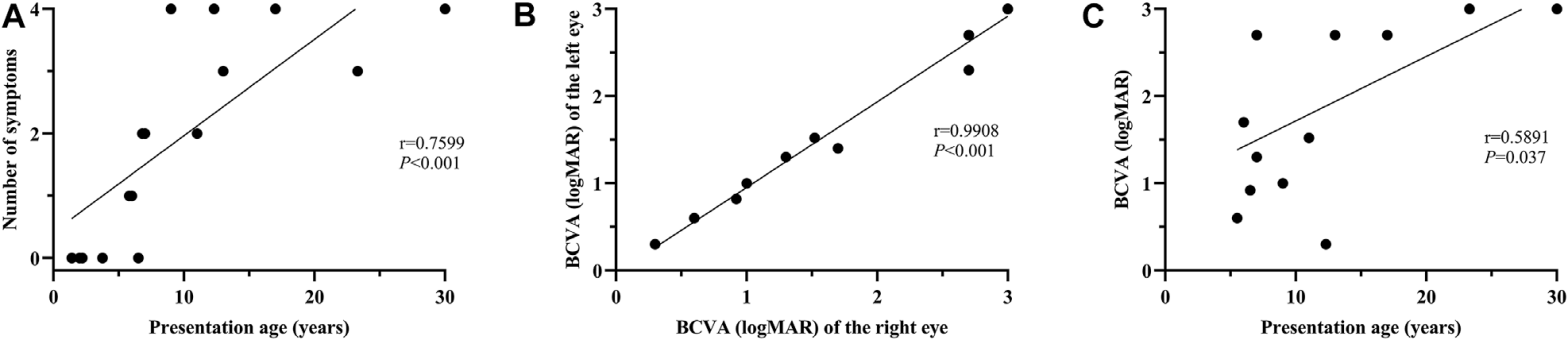
Correlation analysis between the number of systemic symptoms and age at presentation; between BCVA and age at presentation. **(A)** A significant positive correlation is evident between the number of systemic symptoms and presentation age (r = 0.7599, *p* < 0.001). **(B)** Binocular symmetry of BCVA in these patients. A strong correlation is detected between the BCVA of the right and left eyes (r = 0.9908; *p* < 0.001). **(C)** A moderate correlation is found between BCVA and age at presentation (r = 0.5891, *p* = 0.037).

No systemic symptoms were observed in any of the patients younger than 5 years. Acanthosis nigricans was noted at 5.8 years of age, followed by hearing loss at 6.0 years. The endocrine or/and metabolism problems and hearing loss were observed in all patients older than 6.8 years, except for patient 0191552, whose hearing records were unavailable. Hepatic dysfunction, often manifested as elevated transaminase, developed as early as 6.8 years of age and was found in all patients over 12.3 years old, except for patient 0191834. By contrast, renal dysfunction was uncommon in this cohort of patients and occurred later than 9 years of age. No DCM was observed in this cohort. Some patients also demonstrated hypogonadism, featured by small testes (078003), a small uterus and polycystic ovary (0191834), precocious puberty (010647), endocardial fibroelastosis (0191552), cognitive impairment (078021), scoliosis (010737), and brachydactyly (0191834). A significant correlation was detected between the systemic symptom numbers of the patients and their ages (*p* < 0.001) (Figure 2A).

### 3.4 Ophthalmic features

All the patients experienced photophobia and/or visual defects during infancy or in their early childhood (less than 5 years of age), except for patient 010737. The median onset age for ocular symptoms was 0.9 years (range: 0.3–6.0). Nystagmus was observed in 88.2% of the patients. For the 13 patients whose BCVA was available, the median BCVA of the patients younger than 10 years was 1.30 logMAR (range: 0.60–2.70), while the median BCVA of the patients over 10 years was 2.70 logMAR (range: 0.30–3.0). Good binocular symmetry of the BCVA was observed in the patients with a strong correlation coefficient for BCVA (r = 0.9908; *p* < 0.001) (Figure 2B). A moderate correlation was perceived between BCVA and the patient’s age at the last examination (r = 0.5891, *p* = 0.037) (Figure 2C).

### 3.5 Typical retinal features

**FIGURE 3:**
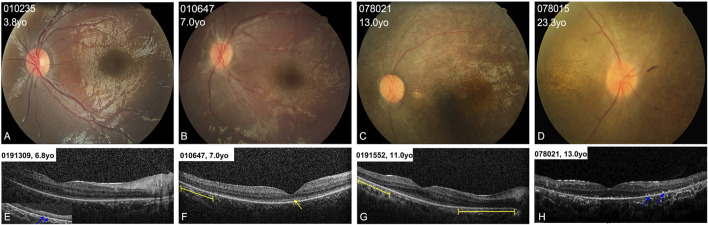
Colored fundus (CF) photographs and SD-OCT images of patients with typical retinal features. **(A)** CF images of patients younger than 5 years old (yo) show blunted foveal reflex and subtle RPE changes in the mid-periphery area. **(B)** CF photographs of patients aged from 5 to 10 years old show blunted foveal reflex, attenuated retinal vessels, and mild tapetoretinal degeneration in the mid-peripheral retina. **(C,D)** CF images of patients older than 10 years of age display a waxy-yellow optic disc, severe vascular attenuation, macular atrophy, and extensive retinal atrophy and clumping pigments in the mid-periphery area. **(E–H)** OCT scans of four patients show loss of the interdigitation zone, and coarse ellipsoid zone (EZ) in the macula (yellow arrow), hyperreflective substance on RPE (blue arrows) in the peripheral retina, and defects of the EZ in the paramacular region (yellow lines).

All the patients showed typical fundus changes, except for patients 010737 and 010809. The patients younger than 5 years (including 5 years old) showed normal optic discs and blood vessels, a blunted foveal reflex, and mild RPE changes in the mid-periphery area (Figure 3A). The probands aged between 5 and 10 years presented with a blunted foveal reflex, attenuated retinal vessels, and tapetoretinal degeneration (TD) in the mid-peripheral retina (Figure 3B). The patients older than 10 years manifested a waxy-yellow optic disc, severe vascular attenuation or white line, macular atrophy, and extensive retinal and RPE atrophy, with clumping pigments in the posterior pole or mid-periphery region (Figures 3C,D). SD-OCT scans of the patients aged between 5 and 15 years displayed relatively normal foveal morphology but a loss of the interdigitation zone (IZ), a coarse ellipsoid zone (EZ) in the macula and paramacular regions, hyperreflective substances in the RPE and all retinal layers, and a mild epiretinal membrane (Figures 3E–H).

### 3.6 Atypical retinal phenotype

**FIGURE 4:**
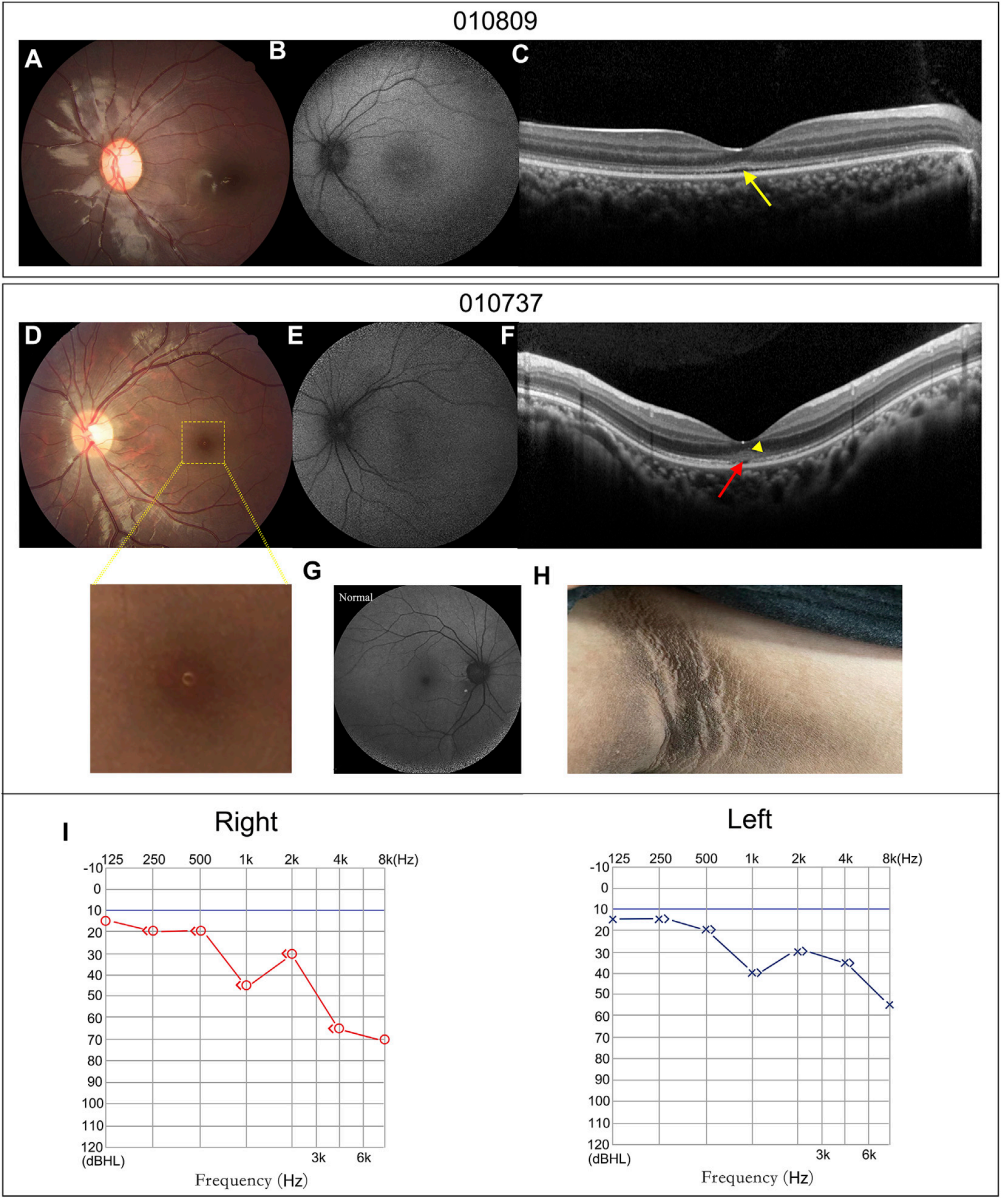
Atypical retinal features of patients 010809 and 010737, and systemic characteristics of patient 010737. **(A,D)** CF photographs exhibit atypical fundus changes with granular-like changes in the macula. **(B,E)** FAF images show loss of the hypo-autofluorescence in the fovea. **(G)** A FAF image of a normal eye. **(C,F)**. OCT scans display coarse EZ (yellow arrow), EZ disruption in the fovea (red arrow) and moderately reflective materials in the outer nuclear layer (yellow triangle). **(H)** Patient 010737 showed acanthosis nigricans in the axillae. **(I)** The PTA of patient 010737 indicates moderate and mild sensorineural hearing loss in the right and left ears.

Patients 010737 and 010809 were the only two patients who did not have nystagmus. Their fundus examinations revealed an almost normal fundus appearance, except for some granular-like changes in the macula (Figures 4A,D). Compared to the normal fundus (Figure 4G), their FAF displayed a loss of the hypo-autofluorescence region in the fovea (Figures 4B,E). Their OCT images revealed slight thinning of the foveal thickness, EZ disruption in the fovea akin to atypical foveal hypoplasia, and moderately reflective materials in the outer nuclear layer of the fovea (Figures 4C,F).

Patient 010737 is worth describing in greater detail. This 12.3-year-old proband had experienced mild visual blurring with photophobia for about 6 years. His BCVA was 0.3 logMAR in both eyes. His ERG recording showed severe dysfunction of the cone cells and moderate impairment of the rod cells. He was clinically diagnosed with CORD. WES revealed an unexpected result, as the proband carried a homozygous c.9542G>A (p.R3181Q) variant in *ALMS1*. His subsequent series of system examinations related to AS disclosed a high-frequency hearing loss (Figure 4I) and acanthosis nigricans was observed in his axillae and neck (Figure 4H). His other systemic components are summarized in Table 2.

Of the 13 patients whose ERG recordings were available, five exhibited severe dysfunction in both rod and cone responses, five showed extinguishing of both cone and rod cell recordings, two (010737 and 010809; both with an atypical retinal phenotype) showed severely impaired cone cell function but moderate rod cell function, and one (010648), aged only 1.4 years, presented only cone dysfunction.

## 4 Discussion

In this study, we performed comprehensive genetic analyses and presented the clinical findings in a Chinese cohort with AS from a tertiary center. We identified a novel missense variant in *ALMS1* and verified that this variant led to abnormal *ALMS1* pre-mRNA splicing. We also observed that the patient harboring the homozygous missense variant showed an atypical retinal phenotype with mild visual defects.

Consistent with previous reports (Marshall et al., 2005; Marshall et al., 2007a; Marshall et al., 2007b; Marshall et al., 2015; Ozantürk et al., 2015; Bea-Mascato et al., 2021), most of the variants (85%, 28/33) detected in the current cohort were clustered in exons 8, 10, and 16, further confirming these three exons as *ALMS1* variant hot spots. The exons 8,10 and 16 are the three largest exons of the *ALMS1* gene, containing 6.1 kilobases (kb), 1.9 kb and 1.2kb, respectively. These three exons occupy 74% (9.2kb/12.5 kb) of the whole coding region, which, in part, makes them the hotspots (Bea-Mascato et al., 2021). All the variants were either truncating variants (non-sense and frameshift indels) or large deletions involving one or several exons. The exceptions were one known splicing variant and one novel missense variant. Bioinformatics analyses suggested that both the reported splicing variant c.7677 + 1G>T and the novel missense variant c.9542G>A (p.Arg3181GLn) triggered cryptic splice donor sites and resulted in exon skipping or truncating. Our RNA analysis results indicated that the missense variant indeed caused an aberrant splicing product and that the consequent frameshift generated a truncated protein p. Ser3082Asnfs*6. This is the first report of a missense variant that can reduce or abolish ALMS1 expression by affecting gene splicing.

Assigning pathogenicity for missense variants of *ALMS1* is rather difficult, as no simple functional assay exists for observing the consequences of this kind of variants (Chen et al., 2017). At present, 19 missense variants related to AS have been recorded in HGMD Professional 2021.4 (Supplementary Table S3). A panel of predictive algorithm analyses demonstrated that four variants affect *ALMS1* splicing and could be defined as likely pathogenic based on the ACMG guidelines and standards. The remaining 15 variants were either benign or of uncertain significance. As the missense variants create cryptic splice sites leading to out-of-frame transcripts, RT-PCR analysis of the ALMS1 mRNA from patients’ tissues or cells or *in vitro* splice assays should be effective ways to observe the effects of missense variants on pre-mRNA splicing.

Clinical diagnosis of AS is still challenging, as its systemic symptoms are age-dependent (Marshall et al., 2007a). In the current cohort, all six probands who were initially diagnosed with AS were older than 7 years of age, whereas the five patients clinically diagnosed with LCA were 7 years of age or younger. When patients only show ophthalmic symptoms, ERG recording might be an important examination to distinguish AS from LCA. Patients with LCA present severely subnormal or non-detectable ERG recordings even in infants (Chacon-Camacho OF and Zenteno., 2015). Our results further proved that more systemic symptoms developed as the patients aged, which contributed to distinguish AS from the simple CORD (Marshall et al., 2007a). AS is categorized as a ciliopathy and needs to be differentiated from other ciliopathies, such as BBS and Joubert syndrome (Aliferis et al., 2012). Polydactyly and cognitive impairment are more common in BBS while hearing problems and T2DM are more frequent in AS (Paisey et al., 2003). In the current study, patient 0191834 was suspected of having BBS, as she had RD, overweight or obesity, and brachydactylia. Brachydactylia is rarely detected in patients with AS but is usually found in patients with BBS(Forsythe and Beales, 2013). Our results reinforced the importance of genetic analysis for patients with atypical AS or patients merely having ophthalmic symptoms. Thus far, no specific and effective therapy is available to stop this progressive multiorgan disorder. However, early diagnosis can slow the progression of some system phenotypes, improve patients’ quality of life, and prolong life expectancy if the affected individuals can obtain timely multidisciplinary care and monitoring (Marshall et al., 2011).

All the patients in this study suffered from ocular symptoms, while the systemic manifestations in our cohort were highly variable. The frequencies of other systemic symptoms were comparable to those reported in previous studies, except for DCM (Marshall et al., 2005; Marshall et al., 2007a; Marshall et al., 2007b; Marshall et al., 2015; Ozantürk et al., 2015; Bea-Mascato et al., 2021), which was not observed in our cohort of patients. In one previously reported Chinese cohort that included 21 patients, DCM was observed in 17% of the patients (Rethanavelu et al., 2020). DCM is not a universal clinical feature, but it is the deadliest feature of AS early on (Bond et al., 2005). Our patients were recruited from an ophthalmic research institution, and patients enrolled from another Chinese ophthalmic research institution in a previous study disclosed that only one of the 11 patients with biallelic null ALMS1 variants had a congenital heart abnormity (Xu et al., 2016).

In the current cohort, 17 patients (90%, 17/19) suffered from early-onset (≤1 year) CORD, with nystagmus or/and severe visual defects, and these are typical ocular findings observed in patients with AS. Their fundus changes were also in line with previous findings (Khan et al., 2015; Dotan et al., 2017; Nasser et al., 2018). The patients under 5 years usually had a relatively normal fundus appearance with blunting of the foveal reflex and mild TD in the peripheral retinal region, whereas patients over 5 years displayed progressive retinal degeneration as they aged. Unfortunately, we did not obtain OCT scanning for the youngest patients. OCT scanning of the patients between 6 and 15 years showed obvious defects in the RPE and outer photoreceptors, even in the mid-peripheral regions. This was consistent with the findings described by Dotan et al. (2017), but our patients all had a relatively normal foveal contour. Our patients’ ERG recordings displayed recordings ranging from severely diminished cone function (age 1.4 years) to severely diminished cone and rod functions (age 2–7 years), and finally to extinguished cone and rod responses (age 5.5–30 years), and these changes were correlated with the ages of the patients at examination.

In the current cohort, only two patients (patients 010737 and 010809) did not display nystagmus. These two patients had similar fundus appearances, but their onset age and the extent of their visual defects differed. Patient 010809 carried biallelic null variants, whereas patient 010737, who harbored a homozygous missense variant, displayed a later onset age (6 vs. 2 years) and better BCVA (logMAR 0.3 vs. 0.8). One recent study reported that a 14-year-old female carrying a compound heterozygous non-sense *ALMS1* variant exhibited an atypical retinal phenotype (Mauring et al., 2020). That case did not show nystagmus, with BCVA logMAR 0.6, and her fundus alteration was similar to that of patients 010737 and 010809.

Patient 010737, carrying the homozygous missense variant, provided a chance for genotype and phenotype correlation analysis. Our RNA analysis for this missense variant indicated that it led to abnormal splicing, which was predicted to trigger NMD; however, a weak normal splicing product was present. We speculated that the mild visual defects might be related to the small amount of normal ALMS1 expression observed in this patient’s lymphocytes. We were unable to calculate the normal intensities due to the lack of a quantitative assay for the RT-PCR products. Furthermore, the results derived from the patient’s lymphocytes may not completely reveal the *in vivo* state of the retina.

Our study had some limitations. One was its retrospective nature, and another was that systemic recordings were incomplete for some patients, and the systemic examinations and evaluations were performed at different hospitals. In addition, the number of patients enrolled in our study was small.

## 5 Conclusion

In conclusion, our findings expand the pathogenic variant spectrum of *ALMS1*. This is the first report of a novel missense variant considered to be pathogenic because it results in aberrant pre-mRNA splicing. Patients with AS might demonstrate varied clinical spectra, and the presence or absence of a particular phenotype was age-related. Patients with missense variants displayed a mild atypical retinal phenotype. Genetic analysis is therefore crucial for the early and accurate diagnosis of patients with atypical AS.

## Data Availability

The data presented in the study are deposited in the Sequence Read Archive (SRA) repository, accession number PRJNA910647.
